# Cost-effectiveness of atezolizumab versus pembrolizumab as first-line treatment in PD-L1-positive advanced non-small-cell lung cancer in Spain

**DOI:** 10.1186/s12962-023-00417-z

**Published:** 2023-01-16

**Authors:** Dolores Isla, Marta Lopez-Brea, María Espinosa, Natalia Arrabal, Diego Pérez-Parente, David Carcedo, Reyes Bernabé-Caro

**Affiliations:** 1 Hospital Clínico Universitario Lozano Blesa, Zaragoza, Spain; 2 Hospital Universitario Marqués de Valdecilla, Santander, Spain; 3 Hospital Regional Universitario de Málaga, Málaga, Spain; 4 Roche Farma S.A, Madrid, Spain; 5 Hygeia Consulting, Madrid, Spain; 6 Hospital Universitario Virgen del Rocío, Sevilla, Spain; 7 Universidad de Sevilla, Sevilla, Spain

**Keywords:** Atezolizumab, Cost-effectiveness analysis, IMpower110 trial, Non-small cell lung cancer, PD-L1 expression, Pembrolizumab

## Abstract

**Background:**

Atezolizumab has recently been approved for first-line treatment of high PD-L1 expression metastatic Non-Small-Cell Lung Cancer (NSCLC) patients with no EGFR or ALK mutations, on the basis of the IMpower110 trial. This study aims to estimate the cost-effectiveness of atezolizumab compared with pembrolizumab among these patients in Spanish settings, based on the results of the two cut-offs of the IMpower110 study.

**Methods:**

A three-state partitioned-survival model was adapted to Spanish settings to calculate health outcomes and costs over a lifetime horizon. Clinical data for atezolizumab were collected from the interim and the exploratory results (data cut-off: Sept’18 and Feb’20, respectively) of the IMpower110 trial while a network meta-analysis was used to model pembrolizumab treatment. Utility data were collected from the trial. Direct medical costs were considered based on resources identified by experts. Costs and outcomes were discounted at 3% per year. Health outcomes were expressed as cost per Life Year (LY) and cost per Quality-Adjusted Life Year (QALY). Both deterministic and probabilistic sensitivity analyses were performed to assess the robustness of results.

**Results:**

Over a lifetime horizon, the incremental results showed that atezolizumab generated similar health outcomes (LYs and QALYs) to pembrolizumab, with minimal differences depending on the cut-off used (+ 0.70 and + 0.42 LYs and QALYs with Sept’18 cut-off and − 0.80 and − 0.72 LYs and QALYs with Feb’20 cut-off). However, for both cut-offs, atezolizumab produced meaningfully less costs than pembrolizumab (€ − 54,261 with Sept’18 cut-off and € − 81,907 with Feb’20 cut-off). The sensitivity analyses carried out confirmed the robustness of the base-case results.

**Conclusions:**

The cost-effectiveness analysis, comparing the two cut-off of IMpower110, shows that atezolizumab provides similar health gains to pembrolizumab but at a lower cost for the first-line treatment of metastasic NSCLC patients in Spain.

**Supplementary Information:**

The online version contains supplementary material available at 10.1186/s12962-023-00417-z.

## Background

Lung cancer (LC) captures the world's attention for it accounts for nearly 20% cancer‐related deaths worldwide [1–3]. In Spain, it was responsible for the highest number of cancer deaths in 2020, causing 22,930 deaths (20.3% of all cancer deaths) [4]. Non-small-cell lung cancer (NSCLC) accounts for approximately 85% of all LC with about 70–75% non-squamous histology and 25–30% squamous histology and is frequently diagnosed as locally advanced or metastatic disease with poor prognosis [5–7].

The use of chemotherapy has been the only systemic therapeutic strategy for decades with five-year survival rates of 0–5% in advanced NSCLC patients [8]. Fortunately, the better understanding of the biology of this cancer and the emergence of immunotherapy has expanded treatment options for advanced NSCLC with improvements in survival, in security and with reduced overall toxicity compared to chemotherapy and other classic cancer therapies [9–12]. In this regard, immunotherapy targeting programmed cell death-1 (PD-1) and programmed cell death-ligand-1 (PD-L1) has markedly improved the overall survival (OS) of patients with locally advanced disease and metastatic NSCLC [13–18]. PD-L1 is expressed on tumor cells (TC) and tumor-infiltrating immune cells (IC) [16] and acts by suppressing the anti-tumour immune response [7, 19].

The first-line immunotherapy anti-PD-(L)1 monotherapy strategy has become the new standard of care in locally advanced and metastatic NSCLC patients with high PD-L1 expression (Tumor Proportion Score [TPS]  ≥ 50%) and no targetable mutations (EGFR mutation or ALK translocations wild-type [WT]) [11, 20, 21]. Until now, pembrolizumab monotherapy was the only first-line treatment option for this subgroup of NSCLC patients [17, 20, 21]. However, given the desirability of having therapeutic alternatives in this subgroup of advanced and metastasic NSCLC patients, other treatments such as atezolizumab, a humanised IgG1 monoclonal antibody anti-PD-L1, has demonstrated significant clinical benefit [22]. Atezolizumab, has recently been approved by the EMA as monotherapy for the “first-line treatment of adult patients with metastatic NSCLC with PD-L1 expression ≥ 50% TC or ≥ 10% tumour-infiltrating IC and who do not have EGFR mutant or ALK-positive” [23].

The approval was based on interim analysis of IMpower110, a global, randomized, open-label, phase 3 trial which evaluated the efficacy and safety of atezolizumab as compared with platinum-based chemotherapy in patients with metastatic non-squamous or squamous NSCLC with EGFR and ALK-WT tumors who had not previously received chemotherapy and who had PD-L1 expression (on at least 1% of TC or of tumor-infiltrating IC) [22]. However, atezolizumab treatment resulted in significantly longer OS than platinum-based chemotherapy only among patients with high PD-L1 expression (TC ≥ 50% or IC ≥ 10%*)* [22]. On Sept’18 (median duration of follow-up:15.7 months), interim results of IMpower110 demonstrated statistically significant improvements in OS for patients with EGFR and ALK-WT tumors who had high PD-L1 expression treated with atezolizumab 1.200 mg compared with platinum-based chemotherapy (4 or 6 cycles) once every 3 weeks [22]. Among these patients, the median OS was significantly longer—by 7.1 months—in the atezolizumab group than in the chemotherapy group (20.2 months vs. 13.1 months; Hazard Ratio [HR]:0.59; 95% confidence interval[CI] 0.40–0.89; P = 0.01) [22]. Among these group of patients, the median PFS was 8.1 months in the atezolizumab group and 5.0 months in the chemotherapy group (HR:0.63; 95%CI 0.45–0.88) [22]. On Feb’20 (median duration of follow-up 31.3 months), according to exploratory results, atezolizumab continued to show a numerical OS benefit vs chemotherapy in the high PD-L1 expression WT-population (median OS: 20.2 months vs. 14.7 months; HR:0.76; 95%CI (0.54–1.09) [24]. Regarding PFS, median PFS was longer in the atezolizumab group than in the chemotherapy group in the high PD-L1 expression WT-population (8.2 months vs 5.0 months, respectively; HR = 0.59; CI 0.43–0.81, p = 0.001] [24].

The development of atezolizumab appears as a first-line treatment option in patients with metastasic NSCLC with high PD-L1 expression according to the recent update of the international ESMO guidelines [21]. For this reason, and in a context in which the efficiency of the health care system has to be considered, the aim of this economic evaluation was to assess the cost-effectiveness of atezolizumab versus pembrolizumab, for first-line treatment of advanced NSCLC patients expressing high levels of PD-L1 (≥ 50%) in Spain, based on the results of the two cut-offs of the IMpower110-study.

## Methods

It should be noted that a panel of expert oncologists validated the model assumptions and parameters introduced for the economic analysis, as well as the clinical feasibility of the results. This article is based on previously conducted studies and does not contain any new studies with human participants or animals performed by any of the authors.

### Model structure

**Fig. 1:**
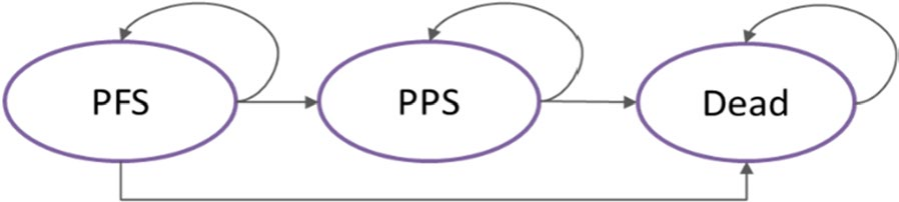
Model structure and transitions. *PFS* progression-free survival, *PPS* post-progression state

A previously developed partitioned-survival model (MS Excel) was adapted to the Spanish settings according to local guidelines and recommendations [25, 26]. The model was used to project health outcomes and costs of NSCLC patients, within three mutually exclusive health states: “progression-free survival” state (PFS) (initial state of patient until progression); “post-progression” state (PPS) (health state after progression) and “death” (absorbing state) (Fig. 1). The length of each model cycle was one week to fit with the various dosage frequencies of interventions. At the end of each 1-month cycle, patients were distributed within these three health states according to area under OS and PFS curves. This model type has previously been used for modeling in metastatic NSCLC in health technology assessments (HTAs) worldwide [27–31].

Over the 30-year time horizon of the analysis (representing patient's lifetime), two hypothetical cohorts of patients (each treated with atezolizumab and with pembrolizumab, in monotherapy) transition through the health states, and for each one the total costs and health outcomes (expressed in Life-Years [LYs], Quality-Adjusted Life Years [QALYs]) were calculated. The incremental cost-effectiveness ratio (ICER) for atezolizumab versus pembrolizumab (expressed as cost per LY/QALY gained) was also calculated.

Costs and outcomes were discounted at 3% per year in line with published recommendations on health technology assessment in Spain [25, 26].

The hypothetical cohort of patients corresponds to all randomised patients with non-squamous or squamous metastatic NSCLC from the IMpower110-study expressing high levels of PD-L1 (TC ≥ 50% or IC ≥ 10%; considered equivalent to TPS ≥ 50%) and no mutations in EGFR and ALK, who had not previously received chemotherapy [22]. The demographic and clinical characteristics of the patient cohort considered in the model were a mean age of 63.73 years and a body surface area of 1.78 calculated according to the Dubois formula [22]. Background mortality has been obtained from the Spanish mortality tables of the National Institute of Statistics [32].

### Clinical inputs

Efficacy data were obtained from the interim results (data cut-off: Sept’18) and from the exploratory (update) results (data cut-off: Feb’20) of the IMpower110-trial. Given that pembrolizumab was not included in the trial, HR from a network meta-analysis (NMA) [33] based in KEYNOTE-024 and KEYNOTE-042 studies [17, 33, 34] were used to extrapolate PFS and OS for pembrolizumab utilizing a random effects (RE) model (HR-RE), available in an additional file (see Additional file 1: Table S1). The NMA authors considered the IMpower110, KEYNOTE-024 and KEYNOTE-042 study cohorts to be comparable [33]. Atezolizumab time-to-discontinuation (TTD) data were considered for both interventions of the analysis, assuming that immunotherapies would have similar treatment durations and TTD is more appropriated than PFS for determining the treatment duration of the immunotherapies. The efficacy of atezolizumab and pembrolizumab monotherapy was assumed to be maintained over the entire time horizon.

In order to extrapolate PFS, OS or TTD data from the IMpower110-trial until the entire time horizon of the economic analysis, parametric curves were fitted to the data reported in the trial with both cut-offs. The goodness of fit results for the parametric functions used to model OS, PFS and TTD for both IMpower110-trial cut-offs was assessed using Akaike information criteria (AIC) and Bayesian information criteria (BIC), available in additional file (see Additional file 1: Tables S2 and S3). A modelling approach utilizing parametric curves from the beginning (cycle 1) to the end of the time horizon of the analysis was used. The clinical plausibility of the clinical trial extrapolations was finally validated on expert opinion and on the basis of visual inspection from literature: phase 2 BIRCH study of atezolizumab [35], Flatiron Health data of pembrolizumab monotherapy, and a 5-year update of KEYNOTE-024 of pembrolizumab monotherapy (31.9% survivals at 5 years) [36]. Therefore, based on AIC and BIC goodness of fit and the clinical plausibility, the best parametric model to extrapolate the PFS and OS data for atezolizumab from both cut-offs beyond was Log-logistic distribution, that predicts 28% OS at 5 years months. For extrapolation of TTD data, Weibull distribution had the best fit.

Extrapolations of Kaplan–Meier (KM) curves for atezolizumab from the results of both cut-offs of IMpower110-trial are available in Additional file 1, along with the coefficient of the models selected as the base case (Additional file 1: Figure S1 and S2).

Regarding safety, treatment-related adverse events (AE) of grade ≥ 3 with an incidence  ≥ 2% for both comparators were incorporated into the model. The incidences of each AE were identified from phase-III clinical trials: IMpower110 (both cut-offs) [22, 24] for atezolizumab and a pool of KEYNOTE-024, KEYNOTE-042 [17, 34] for pembrolizumab. All AE impacts only in terms of costs and not on quality-of-life.

Utility values derived from the EuroQoL-5 Dimensions, 3 Levels (EQ-5D 3L) results of the phase III IMpower110-trial were used in the analysis [22]. For the base case, time on treatment from the IMpower110-trial was used to implement the utility approach in function of time (on/off treatment approach).

### Cost inputs

The analysis was performed from the Spanish Health System perspective, so only direct medical costs (in 2020 euros) were assessed.

Drug acquisition costs include the costs of compared treatments (atezolizumab and pembrolizumab) and the cost of subsequent treatments once patients experience disease progression and transition to ‘PPS’ state. Table 1 shows the distribution of subsequent drugs after the treatment with immunotherapy strategies regardless of whether the therapy was with atezolizumab or pembrolizumab. Administration costs associated with intravenous drugs (211€), obtained from esalud database [37], are also included.

**Table 1:** Distribution of subsequent treatments after treatment with immunotherapies

	Distribution	Source
Chemotherapy		
Pemetrexed	60%	Expert opinion
Gemcitabine	10%	
Carboplatin	45%	
Cisplatin	20%	
Paclitaxel	5%	
Docetaxel	10%	
Vinorelbine	30%	
Targeted therapy		
Bevacizumab	5%	Expert opinion
Nintedanib	5%	

All drug costs were expressed as the ex-factory price considering the corresponding deductions according to royal-decree law 08/2010 [38, 39] where appropriate (see Additional file 1: Table S4). For drugs where the dose is dependent on body weight or body surface area (demographic characteristics described at the end of the model structure section), vial optimisation is assumed.

AE management costs were obtained from the literature [40, 41] or from the Ministry of Health’s publication of Grupos Relacionados por el Diagnostico (GRDs).

Concerning to costs related to disease management while patients are in ‘PFS’ and ‘PPS’ states, the use of resources (visits, tests etc.) was validated by panel of experts and the costs associated to were obtained from a Spanish healthcare cost database [37]. In this regard, the model also included the costs associated with terminal care received by the patient prior to death. These costs are computed as one-off costs (only once, not every model cycle) when patients transition to the ‘death’ state (€ 13,779.95 [42]).

Main model inputs including clinical and costs inputs were shown in following Table 2.

**Table 2:** Model inputs

Model inputs	Base case value	Source
Efficacy parameters^a^		
PFS	Atezo: Log-logistic distribution Pembro: Relative treatment effect HR-RE model	[17, 22, 33, 34]
OS		
TTD	Atezo: Weibull distribution Pembro: Weibull distribution	[22]
Frequencies of AE grade ≥ 3 with incidence ≥ 2% (Atezo/Pembro)		
Hyponatraemia	2.80%/0.00%	[17, 22, 34]
Diarrhoea	0.00%/3.90%	
Pneumonitis	5.61%/3.25%	
Pyrexia	3.74%/0.00%	
Hyperkalaemia^b^	1.87% or 3.74%/0.00%	
Severe skin reaction	0.93%/5.19%	
Utility values (on/off-treatment)		
PFS state	0.76	[22]
PPS state	0.69	
Frequency of disease management resources (PFS/PPS state)		
Outpatient visit	17/19–20 per year	Expert opinion
GP visit	6/8–9 per year	
Hospital nurse visit	4–5/5–6 per year	
Primary care nurse visit	6/8–9 per year	
Chest CT scan (and others)	4/5 per year	
Radiography	1 per year	
AEs unit costs (€,2020)		
Hyponatraemia	4.831,03 €	GRD_APR (weighted severity level of minor and major.2015)
Diarrhoea	1.108,70 €	[41]
Pneumonitis	3.897,50 €	GRD_AP (Weighted 89, 90.2015)
Pyrexia	830,746 €	[41]
Hyperkalaemia	4.831,03 €	GRD_APR (Weighted severity level of minor and major.2015)
Severe skin reaction	2843,81 €	[41]
Disease management unit costs (€,2020)		
Outpatient visit	88,38 €	[37]
GP visit	22,81 €	
Hospital nurse visit	26,99 €	
Primary care nurse visit	21,15 €	
Chest CT scan (and others)	133,56 €	
Radiography	36,20 €	

*Atezo* atezolizumab, *pembro:pembrolizumab*, *PFS* progression-free survival *OS* overall survival, *TTD* time to discontinuation, *AE* adverse events, *GP* general practitioner, *CT* computerised tomography, *GRD *grupos relacionados con diagnósitco, APR all patient refined, *AP* all patient

^a^All the extrapolations are fully parametric

^b^The incidence of all AEs with atezolizumab is the same for both cut-offs of IMpower110-study, with the exception of hyperkalaemia, where the incidence is reflected for the Sept’18 and Feb’20 cutoff, respectively

### Sensitivity analyses

In order to assess the uncertainty of the variables used in the model and to determine the robustness of the results obtained, both deterministic (scenario and univariate) and probabilistic sensitivity analyses (PSA) were performed, with the results of the two cut-offs of IMpower110-study.

Scenario analyses assessed the impact on the ICER of alternative scenarios to the base case, modifying some assumptions, or exploring methodological alternatives.

- *Time horizon*: 10 and 20 years, instead of lifetime (30 years).
- *Alternative parametric curves*: log-normal distribution for SLP and for SG and gamma distribution for TTD.
- *Duration of treatment*: Until progression, assuming the PFS curve used in the model as an approximation.
- *Indirect comparison (NMA)*: HR-Fixed-Effects (HR-FE) model instead of HR-RE model.
- *Utilities:* Two sets of utility values derived from the phase III IMpower110-trial in function of progression status (pre-progression and after progression approach) and in function by time to death (proximity to death approach) instead of in function by time on treatment. Also, disutilities associated with AE were used.

Univariate analysis (one-way sensitivity analysis): Some model variables were individually modified by 10% or 20% (depending on the uncertainty associated with the variable) with respect to the base case.

PSA: 1000 simulations were performed using the Monte-Carlo method [43], simultaneously modifying variables for parametric curves with a multivariate normal distribution (except fractional polynomial NMA functions), utility values with a normal distribution, and frequency of AEs and costs (except pharmacological) with a log-normal distribution.

## Results

### Comparative results

The results of the base case with both cut-offs of IMpower110-study (Sept’18 and Feb’20) are reported in Table 3.

**Table 3:** Case base results

	Atezolizumab		Pembrolizumab		Incremental	
	Cut-off 2018	Cut-off 2020	Cut-off 2018	Cut-off 2020	Cut-off 2018	Cut-off 2020
Costs in ‘PFS’state	€ 105,418	€ 149,213	€ 160,618	€ 227,894	€ − 55,200	€ − 78,681
Treatment (intevention)	€ 95,225	€ 136,135	€ 151,300	€ 216,299	€− 56,075	€ − 80,164
Other healthcare costs	€ 10,193	€ 13,079	€ 9,318	€ 11,595	€ + 875	€ + 1,494
Costs in ‘PPS’state	€ 14,307	€ 12,171	€ 12,778	€ 15,588	€ + 1,529	€ − 3,417
Subsequent treatment	€ 5,681	€ 5,534	€ 5,681	€ 5,534	0	0
Other healthcare costs	€ 8,626	€ 6,637	€ 7,097	€ 10,054	€ + 1,529	€-3,417
Costs ‘end-of-life’	€ 11,176	€ 11,477	€ 11,766	€ 11,286	€ − 590	€ + 191
Total costs	€ 130,901	€ 172,861	€ 185,162	€ 254,769	€ − 54,261	€− 81,907
LYs in PFS	1.93	2.15	1.74	1.79	+ 0.18	+ 0.36
LYs in progression	2.92	2.25	2.41	3.41	+ 0.52	− 1.16
Total LYs	4.85	4.40	4.15	5.20	+ 0.70	− 0.80
QALYs in PFS	0.99	1.43	1.06	1.61	− 0.07	− 0.18
QALYs in progression	2.46	1.73	1.97	2.27	+ 0.49	− 0.54
Total QALYs	3.45	3.16	3.04	3.88	+ 0.42	− 0.72
ICER (€/LY gained)					Dominant*	Less LY*, less cost
ICUR (€/QALY gained)					Dominant*	Less QALY*, less cost

*LY* life years, *QALY* quality-adjusted life years, *ICER* incremental cost-effectiveness ratio, *ICUR* incremental cost-utility ratio

^*^Ratios are not shown as it is a dominant option or a less QALYs/less cost option

The incremental results in the Table 3 show how, in terms of long-term effects expressed in LYs and QALYs, considering the one cut-off or the other, atezolizumab generates more or less LYs and QALYs than pembrolizumab, respectively. This shows that in terms of efficacy both immunotherapies can be considered equally effective, as reported in the recent NMA performed by Herbst et al. [24].

In terms of costs, the incremental results in Table 3 show that for both cut-offs atezolizumab has a lower cost than pembrolizumab (€ − 54,261 with Sept’18 cut-off and € − 81,907 with Feb’20 cut-off).

### Sensitivity analysis

In all the scenarios analysed, the results that show for one cut-off (Sept'18) the dominance of atezolizumab vs. pembrolizumab (more QALYs/less cost) and for the other cut-off (Feb’20) less QALYs but lower cost are maintained. Ratios are not shown as it is a dominant option or a less QALYs/less cost option, as in the base case.

**Fig. 2:**
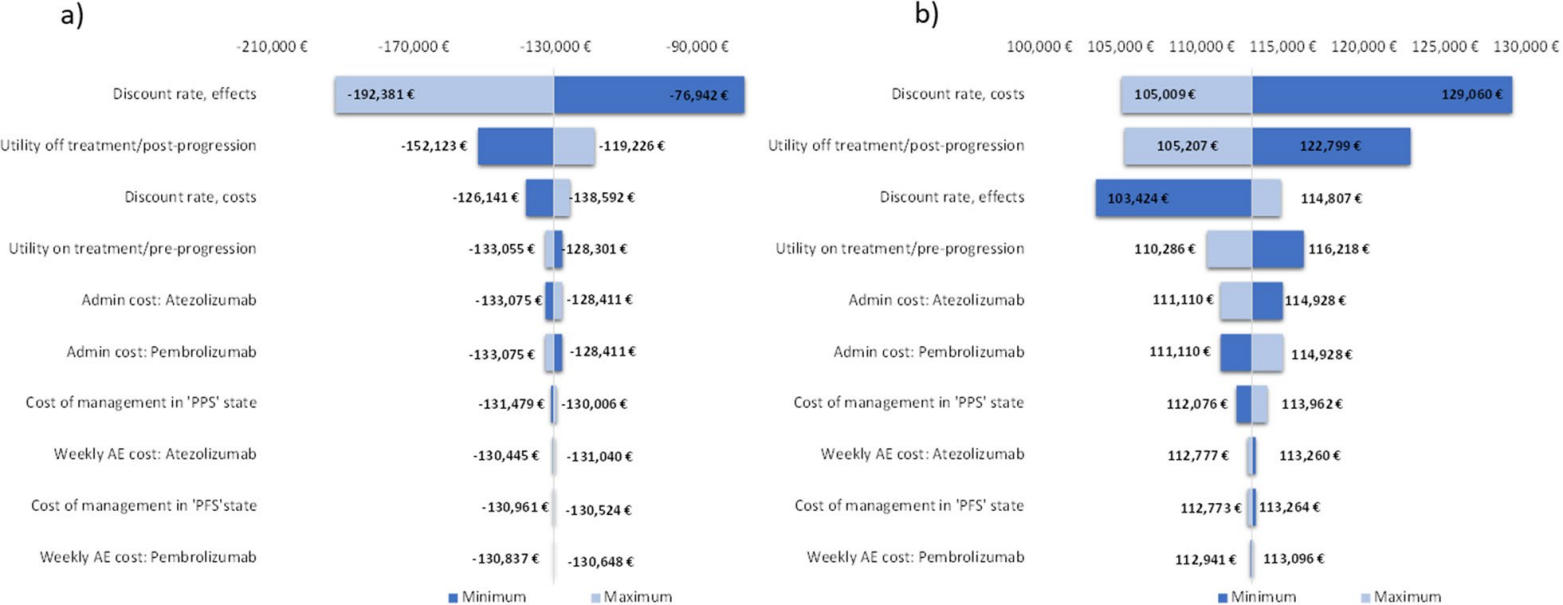
Tornado diagram^a^. *ICUR* incremental cost-utility ratio, *QALY *Quality-Adjusted Life Years, *Admin* administration, *PFS *progression-free, *PPS* post-progression state, *A *adverse events. ^a^ICER values in **a** are shown as negative because they correspond to the ratio of more QALYs at lower cost (dominant), ICER values in **b** correspond to the third quadrant, less QALYs with less cost

The results of the univariate analysis of the base case with the results of the two cut-offs (Sept’18 and Feb’20) are represented in a tornado diagram in Fig. 2, showing the impact of minimum and maximum values of each variable on the base case incremental cost-utility ratio (ICUR), for each cut-off. In the univariate analysis of the results with Sept’18 cut-off, variations on the discount rate of the effects show the highest incidence on the ICUR of the base case while with Feb’20 cut-off, the variable that show the highest impact is the discount rates for costs (Fig. 2). The rest of the variables show the same trend of influence on the ICUR with both cut-offs, and in particular, the cost parameters show a low influence on the ICUR results of both cut-offs (Fig. 2).

Through the PSA, looking at the dispersion of the 1000 simulations performed, the robustness of the cost-effectiveness results of atezolizumab versus pembrolizumab for both cut-offs were evaluated.

**Fig. 3:**
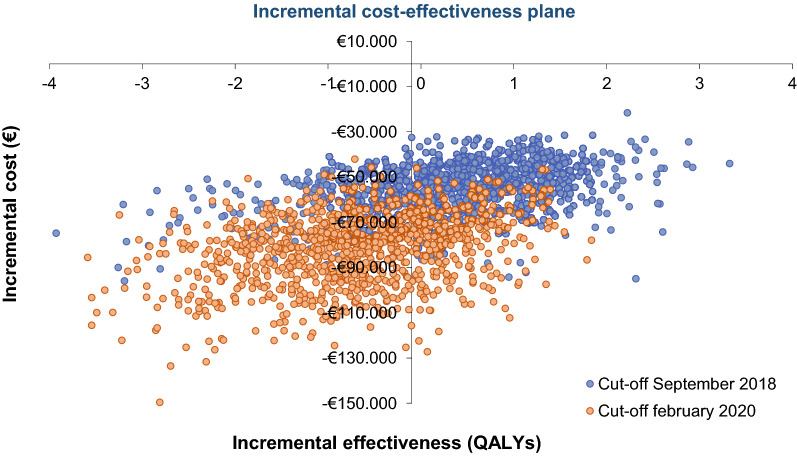
PSA results. Incremental cost-effectiveness plane. The deterministic results of the base case are shown with a dark filled dot. *QALY* quality-adjusted life year

Figure 3 shows the PSA results represented by an incremental cost-effectiveness plane, where on the abscissa axis the incremental QALYs of atezolizumab versus pembrolizumab are plotted, while on the ordinate axis the incremental results in terms of costs are plotted.

All simulations, for both cut-offs, show a lower cost for atezolizumab. In terms of efficacy, the point cloud is generally around zero, which means equal efficacy of atezolizumab and pembrolizumab.

Also, in (Additional file 1: Fig. S3), PSA results are presented in a cost-effectiveness plot, where the results of the 1000 simulations are shown separately for atezolizumab and pembrolizumab, for both Sept’18 and Feb’20 cut-offs.

## Discussion

The present study assessed the cost-effectiveness of atezolizumab versus pembrolizumab for first-line treatment of high PD-L1 (≥ 50%) advanced NSCLC patients in Spain, comparing the results obtained with the interim cut-off (Sept’18) and with the exploratory cut-off (Feb’20) of the IMpower110-study. Efficacy data for atezolizumab were obtained from these cut-offs of IMpower110-study, while pembrolizumab efficacy were obtained from an NMA.

The results of the cost-effectiveness analysis, clearly affected by the IMpower110-study cut-off used (Sept’18 or Feb’20), place atezolizumab as a dominant treatment alternative vs. pembrolizumab, or as an alternative associated with fewer QALYs at a lower cost vs. pembrolizumab, respectively. Although the results from this analysis do not seem very conclusive, this is because the NMA results that indirectly compare atezolizumab vs. pembrolizumab show both treatments to be similar in terms of efficacy, so small differences between the two cut-offs (interim and exploratory analysis) in the IMpower110-study, change the direction of the QALY gain in the model. In this manner, the results of the present analysis reinforce the perception that atezolizumab is as effective as pembrolizumab in delaying disease progression and in extending life. In economic terms, both analyses (cut-offs: Sept’18 and Feb’20) agree that atezolizumab treatment is associated with lower costs than pembrolizumab treatment.

In our analysis, PSA is especially useful for comparing cost and health outcomes for both cut-offs together. Looking at the dispersion of the 1000 simulations, it can be easily observed how the efficacy of pembrolizumab and atezolizumab can be considered broadly similar, with the point cloud of the exploratory 2020 cut-off slightly shifted toward the third quadrant (more QALYs with pembrolizumab) and the point cloud of the 2018 cut-off slightly shifted toward the fourth quadrant (more QALYs with atezolizumab). In economic terms, it can be clearly seen on the cost-effectiveness plane how all simulations show atezolizumab as a lower-cost treatment than pembrolizumab.

Another published study by Chia-Wei et al. (2021) that analyses the cost-effectiveness of atezolizumab versus pembrolizumab as first-line monotherapy in patients with NSCLC and high PD-L1 expression has been found [44]. The analysis, also based on a Markov model but carried out in the United States, concluded that first-line atezolizumab monotherapy had 0.6 LY and 0.47 QALYs gained compared with pembrolizumab monotherapy and was estimated to be cost-effective for their respective health system (ICER $58,841/QALY) [44]. On the other hand, Majem et al. [11] conducted a NMA (six clinical trials and 2111 patients included) to compare effectiveness of both immunotherapy strategies in the first line setting in this type of NSCLC patients and concluded that both atezolizumab and pembrolizumab improve the efficacy outcomes of the patients versus chemotherapy alone. However, according to conclusions of this study, further evaluations to determine the superiority of any specific PD-(L)1 inhibitor are needed.

To understand the differences in the results of the cost-effectiveness analysis in terms of QALYs according to the IMpower110 cut-off, it is worth commenting on the NICE-TA705 for atezolizumab assessment [45], regarding the NMA that feeds into the present analysis [33]. As discussed above, the results of the NMA do not show statistically significant differences between atezolizumab and pembrolizumab for OS, PFS, duration of response and overall-response rate (ORR) and, results from the exploratory cut-off (Feb’20) demonstrate a trend in relative hazards moving in favour of pembrolizumab over time [45]. However, these trends are likely to be the result of a bias. On one side, the larger pembrolizumab trial only has follow-up data in line with the earlier IMpower110 data cut-off (Sept'18). However, according to NICE-TA705 for atezolizumab assessment, additional analyses of the NMA done demonstrated that using the smaller pembrolizumab study that has longer duration of follow up, within the NMA improves the HR slightly for atezolizumab [45]. On the other side, longer follow-up periods in the observational analysis (Feb'20) of the IMpower110-study show plateauing in the chemotherapy arm, resulting in HR for atezolizumab becoming less favorable. This is because more patients with high PD-L1-expressing WT received subsequent immunotherapy in the chemotherapy arm vs. atezolizumab in the observational OS analysis [24]. Indeed, OS HR more strongly favored atezolizumab after adjusting for the effect of subsequent cancer immunotherapy using the RPSFT method [24]. These points indicate that differences in follow-up durations between pembrolizumab and atezolizumab studies lead to results being very similar but biased in favour of pembrolizumab. Therefore, depending on the cutoff applied for the cost-effectiveness analysis, the direction of the cost-effectiveness analysis varies (in terms of QALYs). However, it should be recalled that the approved indication for atezolizumab in monotherapy was based on the Sept’18 cut-off (interim analysis).

In addition to the differences in follow-up between the pembrolizumab and atezolizumab studies included in the NMA, our study has some inherent limitations of the theoretical models. First, faced with the use of a lifetime horizon, the long-term clinical benefits were extrapolated by fitting the parametric functions, beyond the observational time of the IMpower110-trial. This approach which may cause bias between the model results and the real situation, is an inevitable limitation of the study. This ties in with another common limitation in economic evaluations, which is the quality of clinical data. In our case, the possible biases that can be found in the NMA comparing IMpower110 and KEYNOTE-024 and KEYNOTE-042 studies are transferred to the cost-effectiveness analysis, although the PSA performed allows us to observe the uncertainty associated with these possible biases. In this way, it would be interesting to perform further NMAs including the final IMpower110 data or more studies evidencing the use of immunotherapies in NSCLC. Lastly, some key clinical cost, such as cost of adverse events which, although obtained from the literature, may sometimes be overestimated. Also, routine use of resources was obtained from a panel of experts rather than through direct observation. However, several sensitivity analyses were performed to minimize these limitations and the potential uncertainty associated, confirmed the robustness of the results obtained. It is worth noting, that the panel of experts validated all assumptions made, the parameters considered, and the results obtained.

Given the lack of cost-effectiveness studies of PD-L1 inhibitors for first-line treatment of NSCLC patients with high PD-L1 expression, as well as the scarcity of recommended therapeutic alternatives in this type of patients, this pharmacoeconomic analysis and the results obtained are of particular interest and importance.

## Conclusions

In conclusion, the cost-effectiveness analysis of atezolizumab versus pembrolizumab with the two cut-offs of IMpower110-study (Sept’18 and Feb’20) shows a similar gain in terms of QALYs and therefore, it can be determined that both therapies are equally effective as first-line treatment in metastatic NSCLC patients expressing high levels of PD-L1 (≥ 50%) and without EGFR and ALK mutations. In this manner, atezolizumab appears to be a first-line treatment alternative in this type of patients, as it shows similar health gains compared to pembrolizumab but at a lower cost in all scenarios analysed in Spain.

In the current healthcare context, the cost savings of atezolizumab compared to pembrolizumab in the treatment of this type of patients, based on similar health gains with both treatments, may lead to greater efficiency of healthcare resources.

On the other hand, it is to be expected that in the future, the development of direct cross-comparison studies between atezolizumab and pembrolizumab will allow for even more robust cost-effectiveness analyses in order to select the best treatment for these patients and ultimately improve their life expectancy and quality of life.

## Supplementary Information

**Additional file 1. Table S1.** HR from the network meta-analysis (pembrolizumab vs atezolizumab). **Table S2.** AIC and BIC for PFS and OS (PDL1 high [TC/IC3] WT Mixed, Mixed Population), cut-off Sept’18. AIC, Akaike Information Criterion; BIC, Bayesian Information Criterion; PFS:Progression-free survival, OS:Overall survival. **Table S3.** AIC and BIC for PFS and OS (PDL1 high [TC/IC3] WT Mixed, Mixed Population), cut-off Feb’20. AIC, Akaike Information Criterion; BIC, Bayesian Information Criterion; PFS:Progression-free survival, OS:Overall survival. **Figure S1**. OS and PFS extrapolation curves (Atezolizumab)–ITT WT-PD-L1 high: IMpower110 (04-Feb-20 cut-off). ITT:Intention to treat; WT:wild type. **Figure S2** OS and PFS extrapolation curves (Atezolizumab)–ITT WT- PD-L1 high: IMpower110 (10-Sep-18 cut-off). ITT:Intention to treat;WT:wild type. **Table S4** Pharmacological cost of the compared first-line treatments. Maint: maintenance, peme: pemetrexed, gem:gemcitabine, * The corresponding discounts according to royal-decree law 08/2010 are included. **Figure S3.** PSA results. Incremental cost-effectiveness plot. QALY:Quality-Adjusted Life Year.

## Data Availability

The datasets generated during and/or analyzed during the current study are available from the corresponding author on reasonable request. Qualified researchers may request access to individual patient level data through the clinical study data request platform (https://vivli.org/). Further details on Roche's criteria for eligible studies are available here (https://vivli.org/members/ourmembers/). For further details on Roche's Global Policy on the Sharing of Clinical Information and how to request access to related clinical study documents, see here: (https://www.roche.com/research_and_development/who_we_are_how_we_work/clinical_trials/our_commitment_to_data_sharing.htm).

## Abbreviations

AE: Adverse events
AIC: Akaike information criterio
BIC: Bayesian information criterio
EQ-5D 3L: EuroQoL-5 Dimensions, 3 Levels
GRDs: Grupos Relacionados por el Diagnostico
HR-FE: HR-Fixed-Effects
HTAs: Health technology assessments
IC: Immune cells
ICER: Incremental cost-effectiveness ratio
ICUR: Incremental cost-utility ratio
KM: Kaplan–Meier
LC: Lung cancer
LY: Life Year
NMA: Network meta-analysis
NSCLC: Non-small-cell lung cancer
ORR: Overall-response rate
OS: Overall survival
PD-1: Programmed cell death-1
PD-L1: Programmed cell death-ligand-1
PFS: Progression-free survival
PPS: Post-progression state
PSA: Probabilistic sensitivity analyses
QALY: Quality-Adjusted Life Year
RE: Random effects
TC: Tumor cells
TPS: Tumor Proportion Score
TTD: Time-to-discontinuation
WT: Wild-type
