# Supplementary material for: Cost-effectiveness of atezolizumab versus pembrolizumab as first-line treatment in PD-L1-positive advanced non-small-cell lung cancer in Spain
Source: Cost Eff Resour Alloc. 2023 Jan 16;21:6. doi: 10.1186/s12962-023-00417-z (PMC9841669; doi:10.1186/s12962-023-00417-z)
Supplement: Supplementary file 1 — Additional file 1. Table S1. HR from the network meta-analysis (pembrolizumab vs atezolizumab). Table S2. AIC and BIC for PFS and OS (PDL1 high [TC/IC3] WT Mixed, Mixed Population), cut-off Sept’18. AIC, Akaike Information Criterion; BIC, Bayesian Information Criterion; PFS:Progression-free survival, OS:Overall survival. Table S3. AIC and BIC for PFS and OS (PDL1 high [TC/IC3] WT Mixed, Mixed Population), cut-off Feb’20. AIC, Akaike Information Criterion; BIC, Bayesian Information Criterion; PFS:Progression-free survival, OS:Overall survival. Figure S1. OS and PFS extrapolation curves (Atezolizumab)–ITT WT-PD-L1 high: IMpower110 (04-Feb-20 cut-off). ITT:Intention to treat; WT:wild type. Figure S2 OS and PFS extrapolation curves (Atezolizumab)–ITT WT- PD-L1 high: IMpower110 (10-Sep-18 cut-off). ITT:Intention to treat;WT:wild type. Table S4 Pharmacological cost of the compared first-line treatments. Maint: maintenance, peme: pemetrexed, gem:gemcitabine, * The corresponding discounts according to royal-decree law 08/2010 are included. Figure S3. PSA results. Incremental cost-effectiveness plot. QALY:Quality-Adjusted Life Year. [file 12962_2023_417_MOESM1_ESM.docx]

**Additional file 1**

**Table S1**: HR from the network meta-analysis (pembrolizumab vs atezolizumab)

|  | Cut-off 2018 | Cut-off 2020 |
| --- | --- | --- |
| PFS | 1,06 | 1,11 |
| OS | 1,13 | 0,87 |

**Table S2**: AIC and BIC for PFS and OS (PDL1 high [TC/IC3] WT Mixed, Mixed Population), cut-off Sept'18

|  | PFS | | | | OS | | | |
| --- | --- | --- | --- | --- | --- | --- | --- | --- |
|  | Arm A (Atezolizumab) | | Arm B (Chemotherapy) | | Arm A (Atezolizumab) | | Arm B (Chemotherapy) | |
|  | AIC (rank) | BIC (rank) | AIC (rank) | BIC (rank) | AIC (rank) | BIC (rank) | AIC (rank) | BIC (rank) |
| *Exponential* | 485,1(7) | 487,8(5) | 475,3(4) | 477,9(1) | 387,9(7) | 390,6(5) | 441,1(1) | 443,6(1) |
| *Weibull* | 482,5(5) | 487,8(5) | 475,5(5) | 480,7(4) | 384,6(3) | 390(3) | 442,7(4) | 447,8(4) |
| *Log-normal* | 476(2) | 481,4(2) | 477(6) | 482,2(5) | 383,9(1) | 389,3(1) | 447,2(7) | 452,4(7) |
| *Gen gamma* | 478(4) | 486(4) | 475(3) | 482,8(7) | 385,7(6) | 393,8(7) | 444,2(6) | 452(6) |
| ***Log-logistic*** | **476,8(3)** | **482,1(3)** | **472,9(1)** | **478(2)** | **384,2(2)** | **389,6(2)** | **441,9(2)** | **447(2)** |
| *Gompertz* | 475,7(1) | 481,1(1) | 477,3(7) | 482,4(6) | 385,3(5) | 390,7(6) | 443,1(5) | 448,2(5) |
| *Gamma* | 484(6) | 489,4(7) | 474,6(2) | 479,7(3) | 385(4) | 390,3(4) | 442,5(3) | 447,7(3) |

*AIC, Akaike Information Criterion; BIC, Bayesian Information Criterion; PFS:Progression-free survival, OS:Overall survival.*

**Table S3**: AIC and BIC for PFS and OS (PDL1 high [TC/IC3] WT Mixed, Mixed Population), cut-off Feb'20

|  | PFS | | | | OS | | | |
| --- | --- | --- | --- | --- | --- | --- | --- | --- |
|  | Arm A (Atezolizumab) | | Arm B (Chemotherapy) | | Arm A (Atezolizumab) | | Arm B (Chemotherapy) | |
|  | AIC (rank) | BIC (rank) | AIC (rank) | BIC (rank) | AIC (rank) | BIC (rank) | AIC (rank) | BIC (rank) |
| *Exponential* | 634,4(7) | 637,1(7) | 539,5(5) | 542(3) | 578,7(7) | 581,4(6) | 537,1(6) | 539,7(4) |
| *Weibull* | 623,2(5) | 628,5(5) | 541,4(7) | 546,6(7) | 573,3(2) | 578,6(2) | 536(5) | 541,1(6) |
| *Log-normal* | 614,4(1) | 619,7(1) | 536(2) | 541,1(2) | 574(4) | 579,3(4) | 531,5(3) | 536,6(3) |
| *Gen gamma* | 616,2(3) | 624,3(4) | 536,4(3) | 544,2(5) | 574,7(6) | 582,7(7) | 533,2(4) | 540,9(5) |
| ***Log-logistic*** | **615,7(2)** | **621(2)** | **531,5(1)** | **536,7(1)** | **573,2(1)** | **578,5(1)** | **529,5(2)** | **534,7(2)** |
| *Gompertz* | 616,8(4) | 622,1(3) | 538,8(4) | 544(4) | 574,2(5) | 579,5(5) | 529(1) | 534,1(1) |
| *Gamma* | 626,5(6) | 631,8(6) | 541(6) | 546,1(6) | 573,8(3) | 579,2(3) | 537,3(7) | 542,4(7) |

*AIC, Akaike Information Criterion; BIC, Bayesian Information Criterion; PFS:Progression-free survival, OS:Overall survival.*

**Figure S1** OS and PFS extrapolation curves (Atezolizumab) – ITT WT-PD-L1 high: IMpower110 (04-Feb-20 cut-off)

*ITT:Intention to treat; WT:wild type*

**Figure S2** OS and PFS extrapolation curves (Atezolizumab) – ITT WT- PD-L1 high: IMpower110 (10-Sep-18 cut-off)

*ITT:Intention to treat;WT:wild type*

**Table S4**: Pharmacological cost of the compared first-line treatments

|  | €/pack | €/mg* | €/administration* |
| --- | --- | --- | --- |
|  |  |  |  |
| Atezolizumab monotherapy: | | | |
| Atezolizumab | 4.489 €  (1200 mg vial) | 3,46 € | 4.152,09 € |
| Pembrolizumab monotherapy: | | | |
| Pembrolizumab | 3.566 €  (100 mg) | 32,99 € | 6597,10 € |
| Pemetrexed / Gemcitabine + platinum (carboplatin or cisplatin): | | | |
| Carboplatin (50%) | 91,68 €  (600 mg vial) | 0,15 € | 101,45 € (with peme)  84,54 € (with gem) |
| Cisplatin (50%) | 22,30 €  (100 mg) | 0,22 € | 29,79 € |
| Gemcitabine | 87,40 €  (2000 mg vial) | 0,04 € | 175,13 € |
| Pemetrexed | 240 €  (100 mg) | 2,22 € | 1.977,04 € |
|  | 1.200 €  (500 mg) maint. |  |  |

*Maint: maintenance, peme: pemetrexed, gem:gemcitabine, * The corresponding discounts according to RDL 08/2010 are included.*

**Figure S3** PSA results. Incremental cost-effectiveness plot

*QALY:Quality-Adjusted Life Year*
